# DNA methylation of the IL6R gene moderates the association between biopsychosocial factors and depression

**DOI:** 10.1038/s41398-025-03596-w

**Published:** 2025-11-22

**Authors:** Reni Merta Kusuma, Mohammad Hendra Setia Lesmana, Vivi Leona Amelia, Muhamad Ansar, Bayu Satria Wiratama, Muhammad Solihuddin Muhtar, Min-Huey Chung

**Affiliations:** 1 School of Nursing, College of Nursing, Taipei Medical University, Taipei, 11031 Taiwan; 2 Department of Midwifery, Faculty of Health, Universitas Jenderal Achmad Yani, Yogyakarta, 55294 Indonesia; 3 Department of Nursing, Faculty of Medicine, Public Health, and Nursing, Universitas Gadjah Mada, Yogyakarta, 55281 Indonesia; 4 Department of Nursing, Universitas Muhammadiyah Purwokerto, Purwokerto, 53182 Indonesia; 5 Eijkman Research Center for Molecular Biology, Research Organization for Health, National Research and Innovation Agency, Cibinong, 16911 Indonesia; 6 Department of Biostatistics, Epidemiology, and Population Health, Faculty of Medicine, Public Health, and Nursing, Universitas Gadjah Mada, Yogyakarta, 55281 Indonesia; 7 International PhD Program in Biotech and Healthcare Management, Taipei Medical University, Taipei, Taiwan

**Keywords:** Diseases, Depression

## Abstract

Biopsychosocial factors substantially increase the risk of depression in adults, and DNA methylation has been implicated as a mechanism through which these factors influence this risk. This study determined whether methylation levels moderate the association between biopsychosocial factors and depression. Using cross-sectional data from the Taiwan Biobank 2016–2017, the study examined the effects of biopsychosocial factors and DNA methylation on depression. The sample consisted of 96 participants aged 30 to 68 years. Depression and biopsychosocial factors were evaluated using self-reported questionnaires. Biopsychosocial factors included biological factors (age, sex, physical illness, body mass index [BMI]), psychological factors (alcohol experience, smoking experience, and exercise habit), and social factors (education, marriage, and dependency). To obtain methylated gene data, the Taiwan Biobank 2016–2017, 65 biological risk genes, and the GSE113725 dataset were intersected, revealing 5 genes and 14 CpG sites potentially associated with depression (*IL2RB-cg02238178*, *IL2RB-cg11558856*, *IL15RA-cg03108606*, *IL15RA-cg07796897*, *IL15RA-cg08676905*, *IL6R-cg25853020*, *IL6R-cg09257526*, *IL6R-cg04715245*, *FTL-cg04385818*, *FTL-cg03039974*, *ZNF614-cg09503196*, *ZNF614-cg25776555*, *ZNF614-cg03293882*, and *ZNF614-cg15684917*). The results indicated that BMI was negatively associated with depression risk (adjusted odds ratio [aOR] = 0.320, 95% confidence interval [CI] [0.117–0.876]), whereas the methylation of *IL6R_cg09257526* increased the risk of depression (aOR = 2.535, 95% CI [1.006–6.391]) and significantly moderated the association between BMI and depression (aOR = 4.687, 95% CI [1.185–18.542]). BMI plays a crucial role in biological factors and together with DNA methylation of the *IL6R_cg09257526* gene contributes to the occurrence of depression in the Taiwanese population.

## Introduction

Depression affects cognitive processes, emotional wellbeing, and physical health [1]. Depression is a major concern among adults and affects individuals of any age, sex, or lifestyle [2]. In 2023, the World Health Organization reported that depression affects approximately 3.8% of the global population, 5% of adults, and 5.7% of individuals aged 60 years or older; and that an expected 280 million individuals worldwide have depression [3]. In particular, adults aged 30 years or older have an increased risk of depression and risk factors include social isolation, financial stress, and chronic health conditions [4]. Over a 10-year period, the incidence of depression in Taiwan increased by 12%. The prevalence of treated depression increased from 1.6% in 2007 to 1.92% in 2016 [5]. These findings indicate the importance of investigating depression in the Taiwanese population. Although various factors contribute to depression, many remain unidentified. Regulation of biopsychosocial dynamics is hypothesized to reduce the prevalence of depression. Thus, the impact of biopsychosocial factors warrants further investigation [6].

Depression is associated with numerous factors, among which biopsychosocial factors play a essential role [7]. Biopsychosocial models serve as comprehensive frameworks that can be used to examine biological, psychological, and social factors contributing to the development and manifestation of both physical and psychological health conditions [8]. The “biological” component includes an individual’s biological, genetic, and physical characteristics [9], such as age [10–12], sex [11–15], physical health status [16, 17], and body mass index (BMI) [18–21]. Recent studies have reported that genetic factors, such as DNA methylation, can affect the onset of depression through epigenetic mechanisms [22, 23]. The “psychological” aspect involves developmental, psychological, and psychopathological factors [24]. Psychological risk factors, such as smoking [25, 26], alcohol consumption [27], and exercise habits [28, 29], are associated with an increased risk of depression. The “social” component focuses on broader environmental factors, including social and cultural surroundings [8]. Social factors, such as education [30–32], marital status [2, 33], and dependency [34], are also associated with depression. Studies have explored how biological factors, such as BMI, may interact with genetic factors, such as DNA methylation, to affect depression [22, 23]. For instance, DNA methylation may modulate the relationship between various biopsychosocial factors and depression, suggesting the presence of a more complex interplay than has been understood to date. Thus, investigating DNA methylation as a potential moderator of biopsychosocial factors can provide new insights into mechanisms underlying depression.

DNA methylation plays a critical role in the development of depression. Global DNA methylation, which regulates gene expression across the genome, is associated with key neurobiological processes, such as stress response, immune function, and inflammation, all of which are implicated in the development of depression [35]. In particular, gene-specific methylation, such as at *IL6R* and *IL15RA*, affects immune and stress responses, thereby influencing mental health outcomes [36, 37]. The methylation of *IL6R* is particularly important because it moderates inflammation associated with depression [38].

DNA methylation is an essential process that inhibits the transcription of repetitive DNA sequences. DNA methylation is involved in various cellular processes, such as X-chromosome inactivation, genomic imprinting, transposon suppression, chromatin structure regulation, retroviral gene silencing, and epigenetic memory maintenance [22]. Changes in DNA methylation might occur in various brain areas, especially the hippocampus and amygdala [39]. Several studies have suggested an association between DNA methylation and depression [22, 40–42]. Hypomethylation, particularly in the hippocampus, can lead to decreased expression of glucocorticoid receptors, a process that is further exacerbated by depression and suicide [39]. A study observed considerably higher hypomethylation in individuals with depression than in those without depression [43]. Another study indicated that epigenetic alterations, including DNA methylation, may contribute to neuropsychiatric disorders. These changes are potentially reversible and could be as important as genetic defects in the pathogenesis of depression [23]. Therefore, understanding DNA methylation is essential because it can either increase or reduce the risk of depression by acting as a moderating factor.

In statistical analysis, moderating effects refer to the role of DNA methylation in modifying the strength or direction of the association between BMI and depression. Specifically, DNA methylation may amplify, attenuate, or even reverse the nature of this association, highlighting its potential regulatory influence on the interplay between biological and psychological factors [44]. Multiple studies have explored the association between biopsychosocial factors and depression [7, 24, 45] but few studies on this topic have focused on the Taiwanese population [46]. Furthermore, some studies have examined DNA methylation in the Taiwanese population, but only few studies have investigated its association with depression [47–50]. A study examined DNA methylation as a moderator in the relationship between sleep quality and depression in young Chinese adults [51]. Another study explored the link between childhood maltreatment and depression during adolescence [52]. However, no studies have investigated DNA methylation as a moderator in the relationship between biopsychosocial factors and depression. Thus, the current study explored DNA methylation as a moderating variable in the relationship between biopsychosocial factors and depression in Taiwan. In particular, this study examined whether biopsychosocial factors can predict depression in this population. In addition, this study determined the extent to which DNA methylation moderates the relationship between biopsychosocial factors and depression. We hypothesized that DNA methylation moderates this relationship, such that individuals with specific methylation profiles would exhibit depression outcomes.

## Methods

### Study design

This cross-sectional study was conducted using data from the Taiwan Biobank collected between 2016 and 2017. This database contains information on biological and lifestyle factors, thus serving as a valuable resource for identifying health risk factors in Taiwan. Recruitment for the Taiwan Biobank is conducted through multiple channels, including media outlets, posters, pamphlets, and websites. Data collection is facilitated through the collaboration of 29 centers across Taiwan. The Taiwan Biobank strictly adheres to regulations governing data protection and privacy [2].

### Sample

Initially, 4888 participants were recruited from the Taiwan Biobank. The Taiwan Biobank only contains data on individuals aged between 30 and 68 years. Participants were included if they had complete data regarding age, sex, weight, height, personal physical illness history, smoking history, alcohol consumption history, exercise habits, education level, marital status, dependency, depression, and DNA methylation. Participants were excluded if they had incomplete data, were undergoing treatment for mental health, or were receiving medication. Finally, 310 eligible participants were identified.

**Fig. 1:**
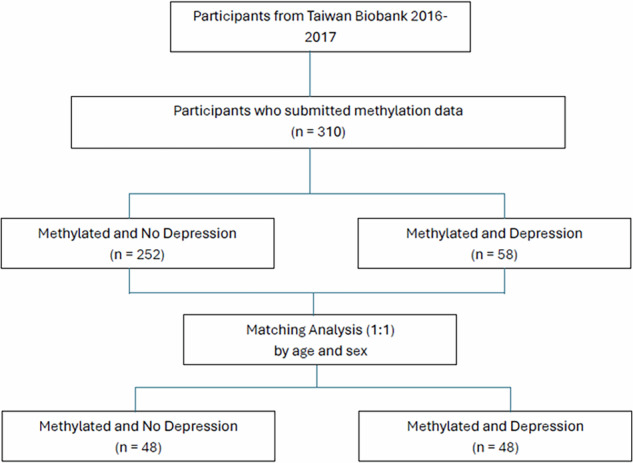
Flowchart of the inclusion procedure. Flowchart illustrating the inclusion procedure and sample size of participants used in the study.

Among the eligible participants, 252 did not have depression, and 58 had depression. Because of the unequal distribution of participants between the two groups [53, 54], a matching analysis was performed. Matching analysis improves study efficiency by reducing the sample size without losing essential information, thereby enhancing the precision of statistical estimates [53]. By minimizing selection bias, matching ensures more accurate and reliable comparisons, increases the study’s validity, and reduces bias [53]. In this study, we conducted matching analysis by manually pairing participants with the same sex and age from both the depression and no depression groups. This process resulted in 48 participants in the depression group and 48 participants from the no depression group. The total number of participants included was 96 (Fig. 1). This study was approved by the Joint Institutional Review Board of Taipei Medical University (approval number: N202306111).

### Measures

#### Biopsychosocial

Biopsychosocial factors were categorized into biological, psychological, and social dimensions on the basis of participants’ characteristics. Biological factors included age and BMI, both recorded as continuous variables, along with sex and physical illness status. BMI was calculated using the following formula: BMI = weight (kg)/height² (m²). This calculation provided a continuous measurement of BMI for each participant [55]. Psychological factors included alcohol experience, smoking experience, and exercise habits. Alcohol experience status was assessed using the question, “Do you currently have a habit of drinking?” Smoking experience status was determined using the question, “Do you currently smoke?” Exercise habits were assessed using the question, “Do you have a habit of exercising regularly?” Social factors included the highest level of education attained, current marital status, and whether the participant lives alone. The variables were categorized as follows: sex (male/female), physical illness (no physical illness/at least one physical illness), alcohol experience (yes/no), smoking experience (yes/no), exercise habits (yes/no), education level (below bachelor’s degree/bachelor’s degree or above), marital status (not currently married/currently married), and dependency (not living alone/living alone).

#### Depression

Depression was measured using a self-reported item from the Taiwan Biobank 2016–2017 questionnaire. Participants were asked the following question: ‘Do you or your family have depression?’ with response options of ‘Yes’ and ‘No.’ This question was presented alongside another item inquiring about the year they were diagnosed with depression by a physician.

#### DNA methylation

DNA methylation data were processed using established quality control protocols to ensure data integrity. Specifically, methylation intensity values were normalized using the normal-exponential out-of-band (noob) method to correct for background fluorescence and ensure the accurate quantification of methylation levels [56]. Batch effect correction was applied using the ComBat method to adjust for technical variations across different sample plates, rows, columns, and chip array positions [49]. These standard procedures have been widely used in DNA methylation studies to ensure high-quality data free from technical biases. To obtain DNA methylation data, several steps were followed. First, data from the Taiwan Biobank (2016–2017) were analyzed using the Limma (Linear Models for Microarray and RNA-Seq data) package in R/Bioconductor framework. In total, 2942 genes in 2016 and 2761 genes in 2017 related to DNA methylation were identified. Second, the Gene Expression Omnibus (GEO) dataset (GSE113725) was used to identify genes related to depression in humans [40]. Analysis was conducted using GEO2R, and 7595 genes were identified. Third, from other study, 65 genes related to biological factors in depressed participants were identified from the Taiwan Biobank 2016–2017 dataset [57].

All the genes were intersected, and the results revealed 5 genes and 14 CpG sites potentially associated with depression. These identified genes and CpG sites were included in the current study: *IL2RB* (*cg02238178* and *cg11558856*), *IL15RA* (*cg03108606*, *cg07796897*, and *cg08676905*), *IL6R* (*cg25853020*, *cg09257526*, and *cg04715245*), *FTL* (*cg04385818* and *cg03039974*), and *ZNF614* (*cg09503196*, *cg25776555*, *cg03293882*, and *cg15684917*).

### Statistical analyses

Descriptive statistics are presented as means and standard deviations (SDs) for continuous data which are analyzed using the independent-samples t-test. Categorical data were compared and statistically analyzed using the chi-square (X²) test and presented frequency and percentage. To standardize numerical data, including age, BMI, and DNA methylation, z-scores were calculated. This step mitigated problems arising from differing variable ranges and minimized the effects of outliers [58, 59]. Collinearity was further assessed by evaluating the variance inflation factor (VIF) within the regression model. The analysis indicated that multicollinearity among the independent variables was unlikely, as all VIF values adhered to the commonly accepted threshold of being below 10 [60]. This approach balances the need for model precision with the inclusion of meaningful variables, ensuring that the findings are robust and interpretable. Furthermore, adjusted odd ratio (aOR) and 95% confidence intervals (CIs) were obtained by performing a conditional logistic regression for depression in association with significant variables including BMI, physical illness, *IL15RA_cg03108606, IL15RA_cg08676905, IL6R_cg09257526, FTL_cg04385818*, and *ZNF614_cg09503196*. Moderation analysis conducted using conditional logistic regression with interaction terms to evaluate whether DNA methylation moderated the relationship between BMI, physical illness, and depression, which was considered statistically significant at *P* ≤ 0.05. Therefore, model reliability was assessed using the conditional logistic regression to identify the best-fitting model, balancing goodness-of-fit and model complexity [61]. In addition, the likelihood ratio test was used to determine whether adding predictors significantly improved model fit. Pseudo R² was calculated as a measure of the model’s explanatory power. Effect sizes were reported as aORs with 95% CIs to ensure the precision and robustness of the estimates [62]. Statistical analyses were performed using STATA with a p value of ≤0.05 considered statistically significant.

The forest plot was used to illustrate the odds ratios (ORs) and 95% confidence intervals (CIs) for the associations between five methylated genes and depression across BMI subgroups (categorized based on lower mean BMI values and a mean BMI greater or equal to the threshold) [63, 64]. This visualization provides a clear representation of effect sizes across different BMI categories, indicating the moderating role of methylated genes in depression.

## Results

Table 1 summarizes the characteristics of 96 adult Taiwanese participants across biological, psychological, social, and methylation factors. Regarding biological factors, the mean age of participants was 51.02 (SD = 11.65) years in both the depression and no depression groups. The majority of participants in this study were female (56.25%); however, the proportions of males and females were balanced between the depression and no-depression groups. This balance indicates that the manual matching process, which paired participants by sex and age, was effectively implemented, ensuring comparability between the two groups. Additionally, 80.21% of the participants reported having at least one physical illness. The mean BMI was 24.55 kg/m² (SD = 3.32). Physical illness and BMI were significantly associated with depression (*P* ≤ 0.05), whereas age and sex were not significantly associated with depression. Regarding psychological factors, most participants were no alcohol drinking (91.67%) and no smoking (63.54%). In addition, 57.29% of the participants reported irregular exercise habits. Alcohol experience, smoking experience, and exercise habits were not significantly associated with depression (*P* > 0.05). Regarding social factors, 52.08% of the participants had a bachelor’s degree or above, and 64.58% of the participants were currently married. Most participants (86.46%) were not living alone. Marital status, education level, and dependency were not associated with depression (*P* > 0.05). The mean ± SD DNA methylation levels at significant CpG sites were as follows: *IL15RA_cg03108606* (0.062 ± 0.053), *IL15RA_cg08676905* (0.128 ± 0.019), *IL6R_cg09257526* (0.310 ± 0.024), *FTL_cg04385818* (0.270 ± 0.158) and *ZNF614_cg09503196* (0.050 ± 0.010). The association between BMI and physical illness variables for Taiwanese and depression severity were shown to present relevant covariates. The results revealed that individuals who have high BMI and at least one has physical illness showed significantly more depression.

**Table 1:** Biopsychosocial and DNA methylation characteristics of participants and the correlation to depression.

Variable	Total	No Depression	Depression	p-value
[Mean ± S.D.] [n (%)]	(n = 96)	(n = 48)	(n = 48)	
Biological Factors				
Age	51.02 ± 11.65	51.02 ± 11.71	51.02 ± 11.71	1
Sex				
Male	42 (43.75)	21 (43.75)	21 (43.75)	1
Female	54 (56.25)	27 (56.25)	27 (56.25)	
Physical Illness				
No physical illness	19 (19.79)	14 (29.17)	5 (10.42)	0.021*
At least one physical illness	77 (80.21)	34 (70.83)	43 (89.58)	
BMI	24.55 ± 3.32	25.34 ± 3.17	23.76 ± 3.31	0.022*
Psychological Factors				
Alcohol Experience				
No alcohol drinking	88 (91.67)	45 (93.75)	43 (89.58)	0.46
Alcohol drinking	8 (8.33)	3 (6.25)	5 (10.42)	
Smoking Experience				
No smoking	61 (63.54)	31 (64.58)	30 (62.50)	0.832
Smoking	35 (36.46)	17 (35.42)	18 (37.50)	
Exercise Habit				
Irregular exercise	55 (57.29)	28 (58.33)	27 (56.25)	0.837
Exercise regularly	41 (42.71)	20 (41.67)	21 (43.75)	
Social Factors				
Education				
Below bachelor’s degree	46 (47.92)	27 (56.25)	19 (39.58)	0.102
Bachelor’s degree or above	50 (52.08)	21 (43.75)	29 (60.42)	
Marriage				
Not currently married	34 (35.42)	14 (29.17)	20 (41.67)	0.2
Currently married	62 (64.58)	34 (70.83)	28 (58.33)	
Dependency				
Not living alone	83 (86.46)	41 (85.42)	42 (87.50)	0.765
Living alone	13 (13.54)	7 (14.58)	6 (12.50)	
Methylation				
IL2RB_cg02238178	0.096 ± 0.013	0.096 ± 0.011	0.096 ± 0.015	0.886
IL2RB_cg11558856	0.135 ± 0.023	0.136 ± 0.023	0.134 ± 0.023	0.635
IL15RA_cg03108606	0.062 ± 0.053	0.049 ± 0.042	0.075 ± 0.060	0.017*
IL15RA_cg07796897	0.059 ± 0.014	0.061 ± 0.014	0.058 ± 0.014	0.209
IL15RA_cg08676905	0.128 ± 0.019	0.134 ± 0.020	0.122 ± 0.017	0.003*
IL6R_cg25853020	0.066 ± 0.011	0.065 ± 0.013	0.067 ± 0.008	0.395
IL6R_cg09257526	0.310 ± 0.024	0.301 ± 0.023	0.318 ± 0.022	0.001*
IL6R_cg04715245	0.064 ± 0.013	0.064 ± 0.013	0.064 ± 0.023	0.983
FTL_cg04385818	0.270 ± 0.158	0.308 ± 0.131	0.233 ± 0.174	0.022*
FTL_cg03039974	0.041 ± 0.009	0.041 ± 0.008	0.041 ± 0.010	0.768
ZNF614_cg09503196	0.050 ± 0.010	0.053 ± 0.007	0.047 ± 0.011	0.003*
ZNF614_cg25776555	0.060 ± 0.009	0.058 ± 0.011	0.061 ± 0.008	0.103
ZNF614_cg03293882	0.063 ± 0.014	0.062 ± 0.014	0.064 ± 0.014	0.438
ZNF614_cg15684917	0.043 ± 0.007	0.042 ± 0.007	0.0433 ± 0.007	0.549

Chi-Squared tests for categorical variables, Independent t-test for continuous variables,

*SD* Standard Deviation, *BMI* body mass index, *IL2R* interleukin 2 receptor subunit beta, *IL15RA* interleukin 15 receptor subunit alpha, *IL6R* interleukin 6 receptor, *FTL* ferritin light chain, *ZNF614* zinc finger protein 614.

*p < 0.05.

Table 2 presents the results of the conditional logistic regression analysis, investigating the association between BMI, 4 methylated genes, depression, and their interactions. The variables that exhibited low multicollinearity, with variance inflation factor (VIF) values below 5 and condition indices within acceptable thresholds were physical illness, BMI, *IL15RA_cg03108606*, *IL15RA_cg08676905*, *IL6R_cg09257526*, *FTL_cg04385818*, and *ZNF614_cg09503196*. These factors included relevant covariates in the moderation analysis. However, *IL15RA_cg03108606* and *FTL_cg04385818* showed severe multicollinearity (VIF > 10), which could potentially destabilize parameter estimates and reduce interpretability in the conditional logistic regression model. Finally, model incorporated only one of the collinear genes (Table 2). The findings reveal that a higher BMI was significantly associated with a reduced likelihood of depression (aOR = 0.320, 95% CI [0.117–0.748], p = 0.027), after controlling for physical illness and methylation levels at *IL15RA_cg08676905, IL6R_cg09257526, FTL_cg04385818*, and *ZNF614_cg09503196*. Higher methylation levels at *IL6R_cg09257526* were significantly associated with an increased risk of depression (aOR = 2.535, 95% CI [1.006–6.391], p = 0.049). Furthermore, methylation at *IL6R_cg09257526* significantly moderated the relationship between BMI and depression, as demonstrated by the significant interaction term (aOR = 4.687, 95% CI [1.185–18.542], p = 0.028). Physical illness was also analyzed; however, none of the results showed significant associations with depression when methylated genes were included as moderators.

**Table 2:** Interaction between BMI and methylated genes to depression.

Variable and interaction	aOR (95% CI)	p-value
BMI	0.409 (0.139–1.197)	0.103
IL15RA_cg03108606	3.387 (1.020–11.244)	0.046
BMI x IL15RA_cg03108606	0.877 (0.417–1.845)	0.73
Physical Illness	2.511 (1.130–5.581)	0.024
IL15RA_cg08676905	0.681 (0.343–1.355)	0.274
IL6R_cg09257526	2.117 (1.061–4.226)	0.033
ZNF614_cg09503196	0.363 (0.135–0.975)	0.045
Pseudo R^2^	0.554	
Log likelihood	−16.326	
BMI	0.419 (0.142–1.236)	0.115
IL15RA_cg08676905	0.685 (0.342–1.372)	0.286
BMI x IL15RA_cg08676905	1.034 (0.413–2.589)	0.943
Physical illness	2.515 (1.125–5.624)	0.025
IL15RA_cg03108606	3.280 (1.021–10.538)	0.046
IL6R_cg09257526	2.102 (1.049–4.213)	0.036
ZNF614_cg09503196	0.374 (0.140–0.999)	0.05
Pseudo R^2^	0.553	
Log likelihood	−16.384	
BMI	0.282 (0.091–0.868)	0.027
IL6R_cg09257526	2.548 (0.926–7.011)	0.07
BMI x IL6R_cg09257526	5.493 (1.195–25.240)	0.029
Physical illness	2.839 (1.145–7.038)	0.024
IL15RA_cg03108606	4.158 (1.080–16.009)	0.038
IL15RA_cg08676905	0.435 (0.158–1.199)	0.108
ZNF614_cg09503196	0.387 (0.134–1.121)	0.08
Pseudo R^2^	0.677	
Log likelihood	−11.824	
BMI	0.476 (0.191–1.186)	0.111
FTL_cg04385818	0.443 (0.181–1.082)	0.074
BMI x FTL_cg04385818	1.075 (0.535–2.158)	0.839
Physical Illness	2.276 (1.083–4.781)	0.03
IL15RA_cg08676905	0.633 (0.311–1.287)	0.206
IL6R_cg09257526	2.171 (1.100–4.286)	0.025
ZNF614_cg09503196	0.366 (0.139–0.966)	0.042
Pseudo R^2^	0.526	
Log likelihood	−17.362	
BMI	0.402 (0.129–1.252)	0.116
ZNF614_cg09503196	0.360 (0.128–1.012)	0.053
BMI x ZNF614_cg09503196	1.280 (0.438–3.741)	0.651
Physical Illness	2.403 (1.071–5.391)	0.033
IL15RA_cg03108606	3.543 (1.019–12.323)	0.047
IL15RA_cg08676905	0.688 (0.343–1.378)	0.291
IL6R_cg09257526	2.131 (1.058–4.291)	0.034
Pseudo R^2^	0.555	
Log likelihood	−16.284	
BMI	0.320 (0.117–0.876)	0.027*
IL6R_cg09257526	2.535 (1.006–6.391)	0.049*
BMI x IL6R_cg09257526	4.687 (1.185–18.542)	0.028*
Physical illness	2.332 (1.057–5.143)	0.036
IL15RA_cg08676905	0.420 (0.158- 1.120)	0.083
FTL_cg04385818	0.431 (0.155–1.197)	0.106
ZNF614_cg09503196	0.364 (0.121–1.093)	0.072
Pseudo R^2^	0.64	
Log likelihood	−13.173	
BMI	0.481 (0.188–1.234)	0.128
IL15RA_cg08676905	0.638 (0.312–1.304)	0.218
BMI x IL15RA_cg08676905	1.083 (0.414–2.831)	0.87
Physical illness	2.296 (1.081–4.878)	0.031
IL6R_cg09257526	2.164 (1.097–4.268)	0.026
FTL_cg04385818	0.449 (0.186–1.078)	0.073
ZNF614_cg09503196	0.371 (0.140–0.980)	0.045
Pseudo R^2^	0.526	
Log likelihood	−17.369	
BMI	0.472 (0.183 - 1.213)	0.119
ZNF614_cg09503196	0.364 (0.135–0.983)	0.046
BMI x ZNF614_cg09503196	1.084 (0.386–3.047)	0.878
Physical Illness	2.232 (1.036–4.805)	0.04
IL15RA_cg08676905	0.630 (0.309–1.281)	0.202
IL6R_cg09257526	2.164 (1.096–4.270)	0.026
FTL_cg04385818	0.445 (0.183–1.084)	0.075
Pseudo R^2^	0.526	
Log likelihood	−17.371	

n = 96. Conditional Logistic Regression;

*p-value < 0.05.

**Fig. 2:**
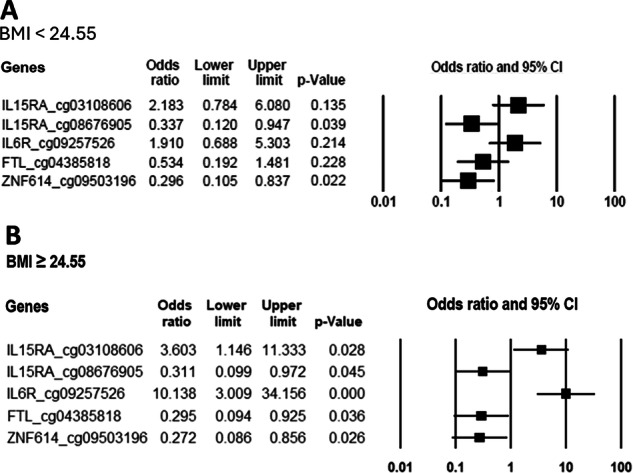
Association between DNA methylation at specific CpG sites and depression risk across BMI categories. Association between DNA methylation at specific CpG sites and depression risk stratified by BMI categories. **A** Participants with lower BMI (<24.55). **B** Participants with higher BMI (≥24.55). BMI was categorized based on the mean value of 24.55 to evaluate differential associations.

Figure 2 illustrates the association between DNA methylation at specific CpG sites and depression risk, classified by BMI categories. BMI was categorized based on the mean value of 24.55 (Table 1), with participants grouped into lower (BMI < 24.55; Fig. 2A) and greater or equal (BMI ≥ 24.55; Fig. 2B) categories. Among participants with a BMI < 24.55, significant associations were identified between the methylation of *IL15RA_cg08676905* (OR = 0.337, 95% CI [0.120–0.947]) and *ZNF614_cg09503196* (OR = 0.296, 95% CI [0.105–0.837]) and depression, while the other 3 genes analyzed did not show significant associations (p > 0.05). Among participants with a BMI ≥ 24.55, all genes significant associations were observed. Methylation at *IL15RA_cg03108606* was linked to a notably high odds ratio (OR = 3.603, 95% CI [1.146–11.333]), suggesting a significant increased risk of depression. Methylation at *IL6R_cg09257526* showed a strong positive association with depression, with a markedly high odds ratio (OR = 10.138, 95% CI [3.009–34.156]). In contrast, methylation at *IL15RA_cg08676905* was associated with a significantly lower odds ratio (OR = 0.311, 95% CI [0.099–0.972]), also indicating a protective effect. Furthermore, methylation at *FTL_cg04385818* (OR = 0.295, 95% CI [0.094–0.925]) and *ZNF614_cg09503196* (OR = 0.272, 95% CI [0.086–0.856]) were both associated with reduced odds of depression, supporting their potential protective roles. These findings suggest that the relationship between DNA methylation and depression may be influenced by BMI, with stronger and more significant associations observed in individuals with a higher BMI.

## Discussion

This is the first study to examine DNA methylation as a potential moderating factor in the relationships between biological, psychological, and social factors and depression by performing matching analysis in a Taiwanese population. The results of the current study revealed that the DNA methylation gene of *IL6R_cg09257526* moderates the strength of the correlation between BMI and depression in the Taiwan population.

Our findings indicated that an increase in BMI was associated with a 0.320-fold reduction in the risk of depression (95% CI [0.117–0.876]; *P* = 0.027; Table 2). Thus, a higher BMI was consistently correlated with a decreased risk of depression, even after controlling for the effect of biopsychosocial factors. In another study, depression was less prevalent among overweight individuals than among underweight individuals [55]. These finding challenges traditional understandings, especially for women in Korea, who experience considerable weight-related pressures and stress due to the high level of societal concern about physical size and weight [55, 65]. BMI does not always correlate with the risk of depression because weight loss efforts can occasionally contribute to an increase in depression [66]. BMI is related to C-reactive protein, and a high level of C-reactive protein is associated with depression, confirming the assumption that other causes of peripheral inflammation (e.g., the interleukin 6 receptor [IL6R]) may affect the development of depression [67].

The methylated gene *IL6R_cg09257526* was significantly correlated with depression (*P* ≤ 0.05; Table 2). The mean depression score for this gene (0.318; Table 1) was higher than that of the control group (0.301; Table 1). IL6R acts as a receptor for the proinflammatory cytokine interleukin-6 (IL-6), enabling it to transmit signals. IL6R is a type 1 cytokine receptor consisting of an IL-6 binding protein and a signal transducing protein [68, 69]. Hypermethylation of *IL6R* was associated with low expression in the no depression group and high expression in the depression group [70]. Other studies have reported that individuals with depression have higher hypomethylation than do those without depression [39, 43]. This finding suggests that increased methylation of *IL6R_cg09257526* can exacerbate depression, supporting its negative effect. This finding is in line with that of a study that identified IL-6 as a potent biomarker for depression, with increased plasma levels of IL-6 genetically predicted to correlate with major depression [71]. Furthermore, another study found that an *IL6R* functional variant could reduce inflammation, and high serum levels of IL-6/IL6R can increase the severity of depression [67]. The current study indicated that an increase in the methylation of *IL6R_cg09257526* significantly increased the risk of depression by 2.535 times (95% CI [1.006–6.391]; *P* = 0.049; Table 2). This finding is consistent with that of a study indicating the correlation of an increased IL-6 level with depression [72]. The pathogenesis of depression is correlated with inflammation, specifically the IL-6/IL-6R pathways; peripheral blood levels of inflammatory markers increased during the acute episodes of depression [73]. Thus, managing IL-6/IL-6R levels in the circulatory system could control both inflammation and depression.

The present study found that the DNA methylation of the *IL6R_cg09257526* gene significantly moderated the relationship between BMI and depression (OR = 4.687; 95% CI [1.185–18.542]; *P* = 0.028; Table 2). A study reported that biopsychosocial factors such as lifestyle are associated with depression and strongly affect DNA methylation patterns [74]. Another study demonstrated that IL-6 moderated the relationships among physical activity, fatigue, and depressed mood [75]. When IL-6 levels are low, the association between physical activity and fatigue is weaker. However, when IL-6 levels are high, physical activity increases fatigue, which is a common symptom of depressed mood [75]. IL-6 can affect the balance of neurotransmitters in the brain (e.g., serotonin, dopamine, and norepinephrine) that are closely involved in mood regulation. Alterations in the level of these neurotransmitters are related to depression [76]. Norepinephrine stimulates the release of IL-6, and IL-6 inhibits the secretion of dopamine and serotonin [77, 78]. Both excessively high and excessively low levels of IL-6 may contribute to mental health problems, such as depression, anxiety, and schizophrenia [79, 80]. IL-6 may cause alterations in the central nervous system [81] and contribute to the development and severity of depression [82]. Furthermore, vagal afferents, circumventricular organs, and brain regions outside the blood–brain barrier can transmit cytokine signals from the periphery to the brain [82], potentially resulting in neurotransmission alterations and subsequent depression [83]. Moreover, in the brain, an increased IL-6 level may cause neuroinflammation, which disrupts normal brain function and affects mood regulation [84, 85]. Higher levels of inflammatory markers and altered neural functioning in several brain regions have been correlated with depression [86]. These genomic and neurobiological pathways might explain why IL-6 is the only inflammatory modifier with sufficient sensitivity to predict the early signs of depression [86].

The strength of the current study lies in its novelty. To the best of our knowledge, no study has examined the role of DNA methylation in Taiwanese individuals with depression considering biopsychosocial factors and investigated DNA methylation as a moderator. The current study performed matching analysis to enhance the robustness of the findings. This technique has been effectively used as an analytical approach in social and community studies [53].

The present study has several limitations. First, the sample size was small, however, another study also included a small number [87], which might affect the generalizability of the results. Thus, caution is advised when interpreting these initial findings, particularly at the CpG level. Second, depression was assessed using self-reported measures that captured participants’ perception of their depression status, based on a prior diagnosis by a healthcare professional. However, self-reported data may be subject to recall bias and could lead to either underreporting or overreporting of depressive symptoms [88, 89]. Third, because the present study had a cross-sectional design, the findings were correlational, and causality cannot be inferred [52].

The findings of this study offer vital implications. Protective factors against depression related to gene methylation were identified. Interventions should focus on helping individuals improve their physical wellbeing. Gene methylation can undergo a reversal process, leading to gene demethylation in the presence of various environmental conditions [22, 23]. Gene methylation is reversible, indicating the importance of adopting a healthy lifestyle [23]. To maintain physical wellbeing, individuals should focus on consuming nutritious food, engaging in regular physical exercise, and ensuring adequate sleep quality [90]. Maintaining a BMI within the normal range is essential for monitoring nutrition status, which can assist in the management of depression [91].

## Conclusion

The findings of this study suggest that biopsychosocial factors, particularly BMI, contribute to the development of depression, with methylated gene of *IL6R_cg09257526* acting as a moderator that exacerbates the condition. In addition, the study highlights the importance of maintaining physical health and achieving an ideal weight as a preventive measure against depression. These findings can assist health-care providers in designing and implementing effective interventions that integrate biopsychosocial factors into mental health treatments. Future research should focus on exploring how depression can be improved through interventions or new strategies, particularly within public health policies addressing BMI status.
